# The Address of Mr. Lawson Tait at the Annual Meeting of the Canadian Medical Association in Montreal

**Published:** 1884-10

**Authors:** 


### Editorial.

---

### THE ADDRESS OF MR. LAWSON TAIT, AT THE ANNUAL MEETING OF THE CANADIAN MEDICAL ASSOCIATION, IN MONTREAL.

In the *Medical News* we find a full report of this remarkable performance of the most remarkable specialist in the very remarkable department of gynecology.

By invitation he appeared before this highly respectable body, and " as the whole aspect of abdominal surgery is at the present moment controversial," according to his mode of viewing professional advancement, he remarks " you need not be surprised if I have made this address somewhat of a polemic, and seemed to have set out with the determination to ignore " the power of judging any question solely upon its merits, and entirely apart from any prejudices, tradition or personal bias."

If Mr. Lawson Tait had been confronted with those whose views and practice were the subject of animadversion on this occasion. so that a rejoinder might have been presented to the same auditory, there might be some excuse for indulging his spleen ; but it cannot fail to strike every reader as indicating a want of manliness and fair dealing, to avail himself of the courtesy of this body of medical brethren to say harsh things of others whose reputation was dear to many of them.

Standing upon neutral ground, he sends back poisoned arrows from his patricidal bow, and hurls his fratricidal tomahawk in front.

But thanks to the triple coat of mail of England's champion ovariotomist and the ægis of honorable rivalry amongst American surgeons, his shafts only recoil upon himself.

Our professional progress is "damned with faint praise" as a fitting return for being "overwhelmed with the kindliest invitations to visit this continent."

It is refreshing to note his egotistic strains in alluding to "the visits of a large number of surgeons from this continent," who came with the belief that they would find he had some secret antiseptic agent, the use of which was the explanation of his success. "Tell it not in Gath," etc.

If he had comprehended "the greatness of the opportunity," in presenting his professional labors to the Association, he would not have forgotten the dignity of his position by descending to personal vituperation and unfair criticism of his colleagues at home and abroad. Yet, notwithstanding all this, many valuable matters introduced in Mr. Tait's address, sustains the high reputation he enjoys in this country; and his special work, when viewed from a purely professional standpoint, entitles him to recognition amongst the foremost gynecologists of the present age.

